# Clinical trends among patients with asthma hospitalized for COVID-19 based on data from a nationwide database: an observational study

**DOI:** 10.1186/s12890-024-02917-x

**Published:** 2024-03-02

**Authors:** Akinari Tsukada, Junko Terada-Hirashima, Jin Takasaki, Hiroshi Nokihara, Shinyu Izumi, Masayuki Hojo, Haruhito Sugiyama

**Affiliations:** Department of Respiratory Medicine, National Center for Global Health and Medicine, Tokyo, 162-8655 Japan

**Keywords:** COVID-19, Asthma, COPD, Intubation, Mortality, Vaccination

## Abstract

**Background:**

While the prevalence of severe cases and mortality rate of coronavirus disease 2019 (COVID-19) appear to be reducing, the clinical characteristics and severity of hospitalized patients with asthma and COVID-19 remain largely unknown. This study aimed to examine the association of asthma with COVID-19 severity and mortality risk.

**Methods:**

Data from the Japanese COVID-19 Registry Database were used to investigate the association between COVID-19 and asthma. This study focused on patients hospitalized for COVID-19 in 690 facilities from January 31, 2020, to December 31, 2022. Multivariate analysis using logistic regression was conducted to assess whether asthma, compared with other conditions, represents a risk factor for mortality and invasive mechanical ventilation after COVID-19.

**Results:**

In total, 72,582 patients with COVID-19 were included in the analysis, of whom, 3731 were diagnosed with asthma. From January 2020 to June 2021, asthma showed no significant association with an increase in mortality (OR 0.837, 95% CI 0.639–1.080, *p* = 0.184) or invasive mechanical ventilation events (OR 1.084, 95% CI 0.878–1.326, *p* = 0.440). An analysis conducted after July 2021 yielded similar results. For patients with asthma, factors such as age, body-mass index, sex, and chronic kidney disease increased the risk of mechanical ventilation. However, non-vaccination status and high blood pressure increased the risk of mechanical ventilation during the second half of the study.

**Conclusion:**

Patients with asthma did not have an increased risk of mortality or mechanical ventilation due to COVID-19. However, patients with asthma had a higher risk of more severe COVID-19 due to factors such as advancing age, elevated body-mass index, chronic kidney disease, and non-vaccination.

**Supplementary Information:**

The online version contains supplementary material available at 10.1186/s12890-024-02917-x.

## Introduction

Coronavirus disease 2019 (COVID-19) is a global pandemic, with over 6 million deaths reported as of July 2023 [1]. The availability of treatments and development of vaccines have helped reduce the risk of severe cases, but the clinical characteristics and course of COVID-19 in patients with asthma remain poorly understood. In a meta-analysis, asthma increased the risk of hospitalization but did not significantly contribute to severe outcomes [2]. In addition, studies from several countries reported that asthma is not a risk factor for severe COVID-19 [3–5]. However, some studies have reported a higher risk of severe outcomes in patients hospitalized due to asthma exacerbation within the past 2 years [6], suggesting potential variability in the severity of asthma and heterogeneity of the population of COVID-19 patients with asthma. Therefore, the factors associated with the severity and mortality of COVID-19 in patients with asthma should be clarified. Furthermore, there is a lack of research that utilizes large-scale national databases to elucidate how being unvaccinated increases the risk of severe asthma at a time when vaccination has become widespread.

This study aimed to assess the association of asthma with COVID-19 severity and mortality risk using a nationwide retrospective multifacility dataset of more than 70,000 individuals admitted across 690 hospitals in Japan.

## Methods

### Ethics considerations

The study was conducted according to the principles of the Declaration of Helsinki and approved by the Ethics Review Committee of the National Center for Global Health and Medicine Hospital (approval number: NCGM-S-004255-02). The requirement for informed consent was waived because the study used an anonymized database.

### Study design and participants

The COVID-19 Registry Japan (COVIREGI-JP) is a research initiative established by the National Center for Global Health and Medicine to comprehensively investigate various aspects of COVID-19. It contains the data of cases of patients diagnosed with COVID-19 who had been admitted to healthcare facilities. This retrospective, multicenter, observational study commenced in March 2020 (https://covid-registry.ncgm.go.jp). The collected demographic and clinical data included date of birth, sex, race, vital signs at admission, symptoms and signs, oxygen therapy status, presence of ventilator support, medication status, and patient outcomes. The study data were collected and managed using REDCap (Research Electronic Data Capture), a secure web-based data capture application hosted at the JCRAC Data Center of the National Center for Global Health and Medicine.

The study focused on patients with COVID-19 who were aged 18 years and above, were admitted to 690 medical facilities in Japan, and had their cases registered in the COVIREGI-JP between January 31, 2020, and December 31, 2022. Patients who had been diagnosed with asthma or chronic obstructive pulmonary disease (COPD) by a physician before hospitalization were categorized into the asthma or COPD group, respectively. Patients who had been diagnosed with both asthma and COPD were assigned to the asthma+COPD group. Additionally, patients who had not been diagnosed with asthma and COPD were assigned to the non-asthma/COPD group and served as the control group.

As the next step in our analysis, we conducted a multivariate analysis using logistic regression to assess whether patients with asthma had higher risks of mortality and mechanical ventilation than those with COPD, diabetes, hypertension, chronic kidney disease (CKD), or cancer. In this analysis, we included age, sex, and body mass index (BMI) as covariates and examined whether the presence, relative to the absence, of each underlying condition increased the risks of mortality and mechanical ventilation. Additionally, we evaluated the presence or absence of a vaccination history as an additional covariate for a later period.

### Clinical and demographic data of the participants

Demographic information, including age, sex, height, weight, and smoking history, and clinical information at admission, including the presence of comorbidities such as hypertension, severe diabetes (patients with diabetic retinopathy, nephropathy, or neuropathy), COPD, renal impairment (indicated by a creatinine level of 3.0 mg/dL, ongoing maintenance dialysis, and post-kidney transplantation), and solid tumors (excluding cases with metastasis). We also calculated the Charlson Comorbidity Index at baseline. Data on vaccination history were collected in July 2021.

The clinical information included the presence of fever, cough, and wheezing at admission and lung parenchymal abnormalities on chest computed tomography scans of patients. Information on the therapeutic agents used for COVID-19 treatment during hospitalization was collected, and the proportions of patients treated with remdesivir and tocilizumab were determined. The steroid dose was retrieved for the patients receiving methylprednisolone at a maximum dose of 1000 mg/day or 1 mg/kg/day or dexamethasone at a dose of 6 mg/day during hospitalization. We also collected data on the duration of hospitalization.

This study identified the patients requiring mechanical invasive ventilation and those who died during hospitalization. The criteria for mechanical invasive ventilation were determined by each facility, with no specific standardized criteria.

Regarding data presentation, a distinction was made between data collected before and after July 2021, when the COVID-19 vaccination had progressed in Japan.

### Statistical analysis

Patient characteristics were compared using the chi-squared test for categorical variables and t-test for continuous variables. Multivariate logistic regression analysis was conducted using patient characteristics such as age, sex, BMI, and comorbidities, including COPD, hypertension, diabetes, renal impairment, and malignant tumors as covariates. Based on this analysis, the odds of death and occurrence of invasive mechanical ventilation were compared for each comorbidity. For data collected after July 2021, the vaccination status was added as a covariate.

Similar to the general patient population, multivariate logistic regression analysis was conducted using the previously mentioned characteristics and comorbidities of patients with asthma as covariates. This analysis aimed to identify the factors contributing to an increase in the odds of death and invasive mechanical ventilation.

To ensure a balance in baseline characteristics, we performed propensity score-matched analysis, allocating patients with and without asthma in a 1:2 ratio. The two groups were matched for covariates such as age, sex, BMI, and comorbidities, including COPD, hypertension, and diabetes. In the second half of the study, vaccination status was also included as a covariate. The occurrence rates of invasive mechanical ventilation and mortality in the matched cohort were assessed using a chi-square test. A significance level of *P* < 0.05 was defined as statistically significant, and R version 4.2.1 was used for the analysis.

## Results

### Baseline characteristics of patients with asthma and COPD

Vaccination data were collected for the period after June 2021. The data are presented separately for the periods from January 2020 to June 2021 and July 2021 to December 2022. The patient demographic data are presented in Table 1. For both periods, patients with asthma had higher proportions of females and younger individuals, whereas those with COPD had a higher proportion of males and older individuals. Patients with asthma had a higher prevalence of obesity than the general patient population and patients with COPD; approximately 15% had BMIs of ≥30 kg/m^2^ for the first and second halves of the duration of the study. In contrast, patients with COPD had lower BMIs. Smoking history revealed that approximately 75% of patients with COPD were current or former smokers. While the influence of age and sex could be considered, there was a consistent trend of a higher prevalence of comorbidities among patients with COPD than among those with asthma. Patients with COPD had a tendency for higher Charlson Comorbidity Index scores. When comparing the first half of the study period with the second half, we observed an increase in Charlson Comorbidity Index scores in both the general patient population and patients with asthma. A higher proportion of patients with COPD had been vaccinated against COVID-19.

**Table 1:** Patient characteristics

Variable	Jan, 2020 – Jun, 2021 (*n* = 46,336)				Jul, 2021 – Dec, 2022 (*n* = 26,246)			
	Non-asthma, COPD	Asthma	COPD	Asthma+COPD	Non-asthma, COPD	Asthma	COPD	Asthma+COPD
	42,819	2302	1087	128	23,905	1429	820	92
Sex female (%)	18,310 (42.8)	1237 (53.8)	139 (12.8)	37 (28.9)	10,896 (45.6)	801 (56.1)	126 (15.4)	16 (17.4)
Age (±SD)	58.54 (20.51)	56.55 (19.71)	73.43 (11.55)	74.20 (10.86)	61.98 (21.83)	57.34 (21.62)	76.67 (11.45)	79.36 (10.09)
Foreigner (%)	1733 (4.2)	67 (3.0)	4 (0.4)	1 (0.8)	698 (3.2)	33 (2.5)	7 (1.0)	3 (3.5)
BMI (±SD)	24.10 (4.69)	25.01 (5.24)	22.93 (3.97)	23.27 (4.37)	23.52 (5.04)	25.14 (5.98)	21.35 (3.97)	21.95 (4.55)
BMI≧30 (%)	3466 (9.9)	312 (15.9)	42 (4.8)	9 (8.7)	2048 (9.9)	205 (16.4)	11 (1.6)	3 (4.1)
Smoking history (%)								
Current	6267 (14.8)	326 (14.2)	258 (23.8)	15 (11.7)	3277 (13.7)	193 (13.5)	168 (20.5)	16 (17.4)
Former	8890 (21.0)	465 (20.2)	594 (54.8)	79 (61.7)	5014 (21.0)	312 (21.9)	457 (55.9)	55 (59.8)
Never	19,571 (46.1)	1124 (48.9)	101 (9.3)	19 (14.8)	11,918 (50.0)	718 (50.3)	104 (12.7)	12 (13.0)
Unknown	7685 (18.1)	382 (16.6)	131 (12.1)	15 (11.7)	3644 (15.3)	204 (14.3)	89 (10.9)	9 (9.8)
Comorbid diseases (%)								
HT	13,589 (31.7)	703 (30.5)	468 (43.1)	3 (2.3)	8596 (36.0)	480 (33.6)	403 (49.1)	47 (51.1)
Severe DM	1025 (2.4)	34 (1.5)	33 (3.0)	4 (3.1)	3892 (16.3)	212 (14.8)	167 (20.4)	12 (13.0)
CKD	741 (1.7)	36 (1.6)	24 (2.2)	4 (3.1)	724 (3.0)	30 (2.1)	21 (2.6)	5 (5.4)
Solid tumor	1466 (3.4)	46 (2.0)	92 (8.5)	8 (6.2)	1081 (4.5)	33 (2.3)	43 (5.2)	7 (7.6)
CCI (±SD)	3.09 (2.5)	2.71 (2.3)	5.98 (2.0)	5.81 (1.8)	3.84 (2.9)	3.04 (2.7)	5.83 (2.4)	5.89 (2.1)
Vaccine history (%)								
Yes (at least once)					10,738 (48.0)	679 (49.5)	497 (65.1)	61 (71.8)
No					7363 (32.9)	486 (35.4)	128 (16.8)	10 (11.8)
Unknown					4287 (19.1)	208 (15.1)	139 (18.2)	14 (16.5)

*COPD* chronic obstructive pulmonary disease, *BMI* body mass index, *HT* hypertension, *DM* diabetes mellitus, *CKD* chronic kidney disease, *CCI* charlson comorbidity index

Table 2 presents the proportion of patients with COVID-related pulmonary abnormalities observed on chest computed tomography (CT) at admission and the presence of specific clinical symptoms. Among the patients who underwent chest CT during hospitalization, the prevalence of pulmonary abnormalities consistent with COVID-related pneumonia among the patients in the asthma and non-asthma/COPD groups were comparable for both periods. However, patients with COPD demonstrated a higher prevalence of these abnormalities. Regarding clinical symptoms, there was a noticeable trend towards a higher proportion of patients with cough and wheezing among those with asthma.

**Table 2:** Findings on admission

Variable	Category	Jan, 2020 – Jun, 2021 (*n* = 46,336)				Jul, 2021 – Dec, 2022 (*n* = 26,246)			
		Non-asthma, COPD	Asthma	COPD	Asthma+COPD	Non-asthma, COPD	Asthma	COPD	Asthma+COPD
		42,819	2302	1087	128	23,905	1429	820	92
Pneumonia on CT (%)	Presence	23,812/30702 (77.6)	1321/1697 (77.8)	716/868 (82.5)	81/96 (84.4)	9712/16359 (59.4)	569/1000 (56.9)	357/596 (59.9)	35/70 (50.0)
Fever (%)	Yes	21,515 (51.2)	1173 (51.1)	615 (56.8)	79 (61.7)	14,448 (60.9)	851 (59.7)	487 (59.6)	52 (56.5)
Cough (%)	Yes	20,918 (49.8)	1435 (62.5)	550 (50.6)	71 (55.5)	14,139 (59.6)	1019 (71.4)	491 (60.2)	63 (68.5)
Wheeze (%)	Yes	577 (1.4)	131 (5.7)	46 (4.2)	14 (10.9)	511 (2.2)	116 (8.1)	48 (5.9)	12 (13.2)

*COPD* chronic obstructive pulmonary disease, *CT* computed tomography

Table 3 shows the treatment details during hospitalization. For both periods, there was a higher prevalence of patients receiving some form of treatment among those with COPD. Among patients with COPD, the use of remdesivir was observed in 30% during the first half of the study and 49% during the second half. Glucocorticoids were more frequently administered to patients with COPD, and higher dosages were also observed. Patients with asthma had a shorter course of COVID-19 (12–14 days) than did those without asthma and COPD. Conversely, patients with COPD tended to have longer courses (approximately 21 days).

**Table 3:** Medication during hospitalization and hospitalization days

Variable	Category	Jan, 2020 – Jun, 2021 (*n* = 46,336)				Jul, 2021 – Dec, 2022 (*n* = 26,246)			
		Non-asthma, COPD	Asthma	COPD	Asthma+COPD	Non-asthma, COPD	Asthma	COPD	Asthma+COPD
		42,819	2302	1087	128	23,905	1429	820	92
Treatment (%)	Yes	21,913 (51.2)	1295 (56.3)	775 (71.3)	92 (71.9)	15,393 (64.4)	961 (67.2)	644 (78.5)	70 (76.1)
Remdesivir (%)	Yes	7387 (17.3)	455 (19.8)	329 (30.3)	37 (28.9)	9292 (38.9)	533 (37.3)	402 (49.0)	42 (45.7)
Tocilizumab (%)	Yes	1272 (3.0)	70 (3.0)	53 (4.9)	4 (3.1)	634 (2.7)	40 (2.8)	21 (2.6)	2 (2.2)
Corticosteroid (%)	Yes	14,318 (33.4)	893 (38.8)	630 (58.0)	74 (57.8)	6817 (28.5)	446 (31.2)	294 (35.9)	29 (31.5)
mPSL1000mg/day		1076 (2.5)	56 (2.4)	55 (5.1)	10 (7.8)	547 (2.3)	18 (1.3)	28 (3.4)	547 (2.3)
mPSL1mg/kg/day		1263 (2.9)	67 (2.9)	51 (4.7)	10 (7.8)	134 (0.6)	6 (0.4)	6 (0.7)	134 (0.6)
DEX6mg/day						5532 (23.1)	350 (24.5)	232 (28.3)	20 (21.7)
Hospitalization days (mean SD)		15.05 (19.40)	14.17 (16.47)	21.20 (23.34)	23.54 (37.45)	15.50 (28.00)	12.58 (18.07)	20.88 (28.65)	17.72 (14.37)

*COPD* chronic obstructive pulmonary disease, *mPSL* methylprednisolone, *DEX* dexamethasone

### Comparison of the rates of mechanical ventilation and mortality in patients with asthma and other diseases

**Fig. 1:**
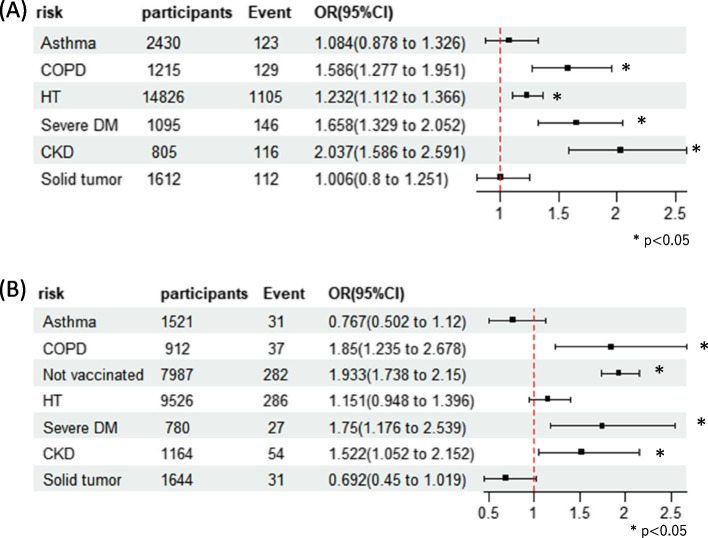
Odds ratio of mechanical ventilation for each comorbidity. **a** January 2020 to June 2021. **b** July 2021 to December 2022. Abbreviations: COPD, chronic obstructive pulmonary disease; HT, hypertension; DM, diabetes mellitus; CKD, chronic kidney disease

**Fig. 2:**
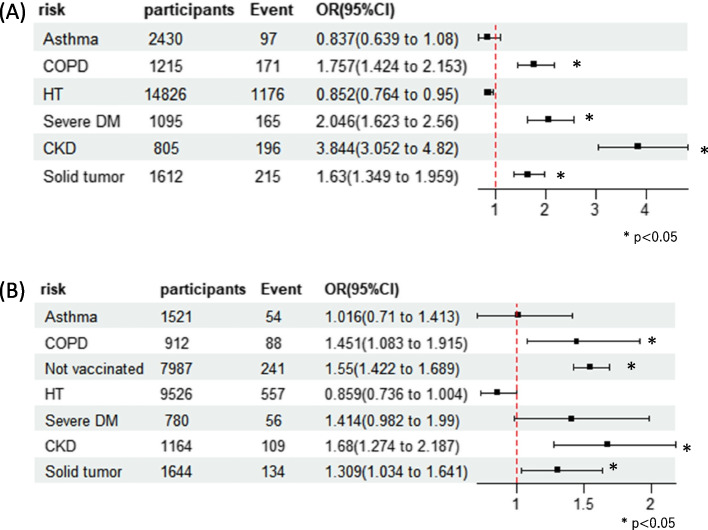
Odds ratio of death for each comorbidity. **a** January 2020 to June 2021. **b** July 2021 to December 2022. Abbreviations: COPD, chronic obstructive pulmonary disease; HT, hypertension; DM, diabetes mellitus; CKD, chronic kidney disease

The odds of invasive mechanical ventilation and death during hospitalization, as the primary outcome measures, are illustrated in Figs. 1A–B and 2A–B. However, the odds of invasive mechanical ventilation did not show a significant increase during the first (OR 1.084, 95% CI 0.878–1.326, *p* = 0.44) or second (OR 0.767, 95% CI 0.502–1.12, *p* = 0.19) half of the study (Fig. 1A–B). In contrast, patients with COPD demonstrated increased odds of invasive mechanical ventilation for the first (OR 1.586, 95% CI 1.277–1.951, *p* < 0.05) and second (OR 1.850, 95% CI 1.235–2.678, *p* < 0.05) halves of the study (Fig. 1A–B). Regarding death outcomes, the odds for patients with asthma did not show a significant increase during the first (OR 0.837, 95% CI 0.639–1.080, *p* = 0.184) or second (OR 1.016, 95% CI 0.710–1.413, *p* = 0.09) halves of the study (Fig. 2A–B). However, for patients with COPD, there was a significant increase in the odds of death for both the first (OR 1.757, 95% CI 1.424–2.153, *p* < 0.05) and second (OR 1.451, 95% CI 1.083–1.915, p < 0.05) halves of the study (Fig. 2A–B). Unvaccinated individuals showed increased odds of invasive mechanical ventilation and death.

In the propensity score-matched analysis, during the first half of the study, there were 2059 patients with asthma and 4117 patients without asthma, matched in a 1:2 ratio. Similarly, in the second half of the study, 1274 patients with asthma and 2547 patients without asthma were matched in a 1:2 ratio. The background characteristics of the propensity score-matched patients are presented in Table S1. During the first half, the rates of mortality were 3.5 and 4.0% in patients with and without asthma, respectively, with no significant difference (*p* = 0.354). Correspondingly, the occurrence rates of invasive mechanical ventilation were 5.4 and 5.2%, without significant differences (*p* = 0.751). In the second half, with vaccination status as a covariate, the rates of mortality were 3.0 and 2.9% in patients with and without asthma, respectively (*p* = 0.914), and the rates of invasive mechanical ventilation were 2.2 and 2.3%, respectively (*p* = 0.846).

### Analysis of the data of patients with asthma

Table 4 shows the results of the analysis of the data of patients with asthma. The study included 2430 participants during the first half and 1521 participants during the second half. During the first half of the study period, significant increases in age and BMI were associated with increased odds of invasive mechanical ventilation and death (OR 1.032, 95% CI 1.019–1.047, *p* < 0.05; OR 1.097, 95% CI 1.058–1.137, *p* < 0.05, respectively). In females, there was a decrease in the odds of invasive ventilation (OR 0.611, 95% CI, 0.404–0.918; *p* < 0.05), whereas CKD was associated with an increase in the odds of invasive ventilation (OR 4.720, 95% CI, 1.731–11.652; *p* < 0.05). Regarding death outcomes, COPD and CKD increased the odds (OR 2.249, 95% CI 1.062–4.471, *p* < 0.05; OR 4.262, 95% CI 1.410–11.3369, *p* < 0.05, respectively). During the second half, when vaccine distribution was more advanced, unvaccinated individuals and those with hypertension had increased odds of invasive mechanical ventilation (OR 1.741, 95% CI 1.058–2.835, *p* < 0.05; OR 3.455, 95% CI 1.367–9.436, *p* < 0.05, respectively). The rate of death was lower during the second half, and only age was associated with increased odds (OR 1.050, 95% CI, 1.026–1.076; *p* < 0.05).

**Table 4:** Asthma patients analysis

Variable	Jan, 2020 – Jun, 2021 (*n* = 2430)		Jul, 2021 – Dec, 2022 (*n* = 1521)	
	OR (95%CI)	*P* value	OR (95%CI)	*P* value
Mechanical ventilation				
Age	1.032 (1.019 to 1.047)	< 0.001*	1.007(0.981 to 1.033)	0.60
BMI	1.097 (1.058 to 1.137)	< 0.002*	1.038(0.978 to 1.094)	0.18
Sex (Female)	0.611 (0.404 to 0.918)	0.018*	0.552(0.235 to 1.236)	0.16
COPD	0.740 (0.274 to 1.664)	0.51	0.943(0.139 to 2.835)	0.94
HT	1.154 (0.747 to 1.782)	0.52	3.455(1.367 to 9.436)	0.011*
Severe DM	0.401 (0.061 to 1.490)	0.24	1.202(0.159 to 5.503)	0.83
CKD	4.720 (1.731 to 11.652)	0.001*	2.482(0.484 to 9.4218)	0.22
Solid tumor	1.175 (0.324 to 3.276)	0.78	0.734(0.040 to 3.859)	0.77
Not vaccinated			1.741(1.058 to 2.835)	0.026*
Death				
Age	1.100(1.076 to 1.127)	< 0.001*	1.050(1.026 to 1.076)	< 0.001*
BMI	1.078(1.016 to 1.141)	0.019*	1.010(0.939 to 1.076)	0.78
Sex (Female)	0.752(0.443 to 1.276)	0.29	0.573(0.281 to 1.149)	0.12
COPD	2.249(1.062 to 4.471)	0.026*	1.384(0.907 to 2.069)	0.12
HT	0.620(0.364 to 1.046)	0.075	1.353(0.456 to 3.490)	0.56
Severe DM	1.421(0.373 to 4.240)	0.56	1.684(0.817 to 3.591)	0.17
CKD	4.262(1.410 to 11.3369)	0.006*	0.577(0.302 to 3.264)	0.61
Solid tumor	1.546(0.490 to 4.033)	0.41	1.319(0.196 to 5.145)	0.73
Not vaccinated			1.814(0.491 to 5.208)	0.31

*p < 0.05

*OR* odds ratio, *COPD* chronic obstructive pulmonary disease, *BMI* body mass index, *HT* hypertension, *DM* diabetes mellitus, *CKD* chronic kidney disease

Consequently, we classified the patients into three age groups (18–44 years, 45–64 years, and ≥ 65 years) to investigate age-specific variations in risk factors. Similar multivariate analyses were conducted (Tables S2 and S3). The number of patients was lower than in the overall analysis, and in asthma patients, the outcomes of death and mechanical ventilation were less frequent, making it challenging to calculate accurate odds ratios. In some instances, we reduced covariates and conducted the analysis. Additionally, in the second period, zero deaths and one case of invasive mechanical ventilation occurred in the 18–44 years age group; thus, the analysis was not conducted. In the first period, among the younger population, increased BMI was associated with a rise in mechanical ventilation. In the middle-aged and older individuals with asthma complicated by CKD or COPD, a noticeable trend of higher odds of mechanical ventilation and death was observed. In the later period, non-vaccinated individuals in the middle-aged group had increased rates of invasive mechanical ventilation and death.

## Discussion

This retrospective, multicenter, observational study involved patients hospitalized for COVID-19 in Japan. It aimed to compare the severity and mortality risk of COVID-19 in patients diagnosed with asthma with those of patients with COPD and other comorbidities. The results showed that patients with asthma did not exhibit an increase in the odds of invasive mechanical ventilation or mortality even after adjusting for background factors, such as age and sex. Similarly, in the propensity score matched-analysis, asthma showed no association with increased events of mortality or invasive mechanical ventilation. Due to the characteristics of the database, data before and after July 1, 2021, were provided separately. However, no increase in the risk of mortality or invasive mechanical ventilation was observed in patients with asthma even on considering vaccination history when analyzing the data. In a specific analysis targeting patients with asthma, an increase in the odds of invasive mechanical ventilation was observed for non-vaccinated patients and patients with hypertension.

As of 2021, reports indicated increased rates of mechanical ventilation and ICU admission due to COVID-19 for patients with asthma without a significant increase in the mortality rate [7]. However, multicenter data from Scotland reported an increase in ICU admissions and deaths, specifically for patients with bronchial asthma [6]. Our analysis, which primarily focused on Japanese individuals, showed no increase in the incidence of invasive mechanical ventilation or the mortality rate. There are several possible explanations for this. First, interracial differences may have played an important role. Genetic studies have shown lower susceptibility to severe COVID-19 in East Asian populations, which may be reflected in our results [8]. Additionally, in the current study, we were unable to collect data on the baseline treatment of asthma patients. However, multiple reports have indicated that inhaled corticosteroids may prevent the worsening of COVID-19 [9, 10], and baseline treatment for asthma may have influenced the results observed in our study.

For individuals with COPD, we observed an increase in the adjusted odds for both mortality and the incidence of invasive mechanical ventilation even after accounting for factors such as age, sex, and vaccination history. This suggests a distinct risk profile from that of bronchial asthma. It is well known that COPD is associated with a higher frequency of comorbidities and a higher mortality rate, relative to asthma [11]. Furthermore, the underlying pathophysiology of COPD, including irreversible airflow obstruction and alveolar structural damage, may be related to the severity of COVID-19.

The analysis targeted 2430 patients with bronchial asthma during the first half of the study and 1521 during the second half. While the occurrence of asthma exacerbation could not be precisely identified in this study, compared to the general population, a higher proportion of asthma patients exhibited wheezing or coughing upon admission. This suggests that these symptoms may serve as indicators of asthma exacerbation. The risk profile, including factors such as advanced age and obesity, during the first half was similar to that previously reported for the general population [12, 13]. Coexisting renal dysfunction has been associated with increased disease severity and mortality rates for both the general population and patients with asthma. Previous reports have shown an association between a lower estimated glomerular filtration rate (eGFR) and increased COVID-19 severity [14]. Given that patients with creatinine levels exceeding 3.0 mg/dL, as well as those receiving dialysis, were included in our study, it is likely that a higher proportion of severe cases of COVID-19 was represented.

During the second half of the study, the number of deaths and severe cases decreased due to the dissemination of drugs and vaccines. However, the odds for the incidence of invasive mechanical ventilation increased in patients with asthma who were not vaccinated and those with concomitant hypertension. While this increase in risk was not observed during the first half, several factors may have contributed to the observed increase in invasive mechanical ventilation in patients with hypertension as the sole comorbidity. The prevalence of hypertension is high among the general population and patients with asthma. Individuals with comorbidities such as CKD, severe diabetes, and COPD demonstrated behaviors such as vaccine acceptance and contact avoidance. However, they may not have been as prevalent when hypertension was the sole comorbidity, and a lower proportion of individuals may have made efforts to mitigate the risk of severe outcomes. Additionally, the regulation of angiotensin converting enzyme 2 expression has been associated with various factors, including obesity, sex, asthma, and hypertension [15, 16], suggesting an influence on the relationship between asthma and hypertension observed in this study. A higher proportion of patients with COPD were vaccinated against COVID-19, which may be attributed to the prioritized vaccination of older adults in Japan. Similar to the general population, vaccination can significantly prevent severe outcomes in patients with asthma.

This study has few limitations. First, it only included hospitalized patients and excluded those treated at home. The observed high prevalence of comorbidities in the cohort according to the baseline Charlson Comorbidity Index suggests the likelihood of hospitalization for a population with multiple comorbidities. These results should be interpreted with caution, as mild cases or those with fewer comorbidities, especially among younger individuals, might have been treated at home, indicating a potential limitation in the generalizability of the study findings. Second, data on the severity of asthma and treatments, such as inhaled corticosteroids, were not collected. Inhaled corticosteroids have been reported to prevent progression to severe outcomes in early-stage COVID-19 [17]. This study used a large-scale registry database designed for COVID-19, and detailed information on the status of complication control and treatment was not collected to allow the enrollment of as many COVID-19 cases as possible in Japan. Nevertheless, risk factors could be identified based on basic patient data, such as age, sex, and comorbidities. An additional limitation is the inability to adjust for baseline characteristics of patients for whom information was not available in the database. Finally, this was a retrospective observational study, and data were collected retrospectively from electronic medical records, which may have resulted in inaccuracies. Nevertheless, our study is a large-scale multicenter study utilizing data from 690 facilities within Japan. The strength of our research lies in the adjustment for factors such as age, sex, comorbidities, and vaccination status, and the comprehensive analysis conducted.

In conclusion, patients with asthma did not show increased severity or mortality risk of COVID-19 relative to patients with comorbid COPD or those without asthma. However, patients with asthma who were older, had higher BMI, or had CKD or those who were not vaccinated had an increased risk of severe outcomes.

### Supplementary Information

**Additional file 1: Table S1.** Characteristics of propensity score-matched patients with asthma. Abbreviations: COPD, chronic obstructive pulmonary disease; BMI, body mass index; HT, hypertension; DM, diabetes mellitus; CKD, chronic kidney disease.

**Additional file 2: Table S2.** Analysis of patients with asthma for occurrence of mechanical ventilation. Abbreviations: COPD, chronic obstructive pulmonary disease; BMI, body mass index; HT, hypertension; DM, diabetes mellitus; CKD, chronic kidney disease.

**Additional file 3: Table S3.** Analysis of patients with asthma for occurrence of death. Abbreviations: COPD, chronic obstructive pulmonary disease; BMI, body mass index; HT, hypertension; DM, diabetes mellitus; CKD, chronic kidney disease.

## Data Availability

The datasets used and analyzed in the current study are available from the corresponding author upon reasonable request.

## Abbreviations

BMI: Body mass index
CKD: Chronic kidney disease
COPD: Chronic obstructive pulmonary disease
COVID-19: Coronavirus disease 2019 (COVID-19)
CT: Computed tomography
