# Supplementary material for: Clinical trends among patients with asthma hospitalized for COVID-19 based on data from a nationwide database: an observational study
Source: BMC Pulm Med. 2024 Mar 2;24:105. doi: 10.1186/s12890-024-02917-x (PMC10909272; doi:10.1186/s12890-024-02917-x)
Supplement: Supplementary file 1 — Additional file 1: Table S1. Characteristics of propensity score-matched patients with asthma. Abbreviations: COPD, chronic obstructive pulmonary disease; BMI, body mass index; HT, hypertension; DM, diabetes mellitus; CKD, chronic kidney disease. [file 12890_2024_2917_MOESM1_ESM.docx]

**TableS1: Characteristics of propensity score-matched asthma patients**

| Variable |  | Jan, 2020 – Jun, 2021 | | |  |  |  |  |  | Jul, 2021 – Dec, 2022 | | | | |  | |
| --- | --- | --- | --- | --- | --- | --- | --- | --- | --- | --- | --- | --- | --- | --- | --- | --- |
|  |  | Non-asthma (n=4117) | Asthma (n=2059) | | SMD |  |  |  |  |  | Non-asthma (n=2547) | | Asthma (n=1274) | | SMD | |
| Sex female (%) |  | 2078 (50.5) | 1068 (51.9) |  | 0.028 | | | | | | | 1148 (45.1) | | 583 (45.8) | 0.014 |  |
| Age (±SD) |  | 56.1 (19.8) | 56.4 (19.2) |  | 0.014 | | | | | | | 62.4 (21.9) | | 62.3 (21.4) | 0.004 |  |
| BMI (±SD) |  | 24.82 (5.17) | 24.88 (5.12) |  | 0.013 | | | | | | | 22.3 (4.5) | | 22.3 (5.0) | 0.007 |  |
| COPD (%) |  | 233 (5.7) | 101 (4.9) |  | 0.034 | | | | | | | 134 (5.3) | | 68 (5.3) | 0.003 |  |
| HT (%) |  | 1300 (31.6) | 641 (31.1) |  | 0.01 | | | | | | | 812 (31.9) | | 417 (32.7) | 0.018 |  |
| Severe DM (%) |  | 71 (1.7) | 35 (1.7) |  | 0.002 | | | | | | | 51 (2.0) | | 31 (2.4) | 0.029 |  |
| CKD (%) |  | 71 (1.7) | 33 (1.6) |  | 0.01 | | | | | | | 68 (2.7) | | 35 (2.7) | 0.005 |  |
| Solid tumor (%) |  | 69 (1.7) | 42 (2.0) |  | 0.027 | | | | | | | 92 (3.6) | | 44 (3.5) | 0.009 |  |
| Vaccine history (%) |  |  |  |  |  | | | | | | |  | |  | 0.035 |  |
| Yes (at least once) |  |  |  |  |  | | | | | | | 1220 (47.9) | | 624 (49.0) |  |  |
| No |  |  |  |  |  | | | | | | | 914 (35.9) | | 459 (36.0) |  |  |
| Unknown |  |  |  |  |  | | | | | | | 413 (16.2) | | 191 (15.0) |  |  |

Abbreviations: BMI, body mass index; COPD, chronic obstructive pulmonary disease; HT, hypertension; DM, diabetes mellitus; CKD, chronic kidney disease
