# Supplementary material for: Clinical trends among patients with asthma hospitalized for COVID-19 based on data from a nationwide database: an observational study
Source: BMC Pulm Med. 2024 Mar 2;24:105. doi: 10.1186/s12890-024-02917-x (PMC10909272; doi:10.1186/s12890-024-02917-x)
Supplement: Supplementary file 2 — Additional file 2: Table S2. Analysis of patients with asthma for occurrence of mechanical ventilation. Abbreviations: COPD, chronic obstructive pulmonary disease; BMI, body mass index; HT, hypertension; DM, diabetes mellitus; CKD, chronic kidney disease. [file 12890_2024_2917_MOESM2_ESM.docx]

**TableS2: Asthma patients analysis (mechanical ventilation)**

| Variable |  | Jan, 2020 – Jun, 2021 | | | Variable | | | | | Jul, 2021 – Dec, 2022 | | | |  |  |
| --- | --- | --- | --- | --- | --- | --- | --- | --- | --- | --- | --- | --- | --- | --- | --- |
|  |  | OR (95%CI) | P value | |  | | | | |  | OR (95%CI) | P value | |  |  |
| Age 18-44 (n=649) |  |  |  |  | |  | |  |  | | |  | |  |  |
| Age |  | 0.966 (0.860 to 1.083) |  | 0.54 | |  | |  |  | | |  | |  |  |
| BMI |  | 1.122(1.001 to 1.238) |  | 0.022* | |  | |  |  | | |  | |  |  |
| Sex (Female) |  | 1.227 (0.221 to 6.839) |  | 0.81 | |  | |  |  | | |  | |  |  |
| HT |  | 3.803 (0.651 to 23.175) |  | 0.13 | |  | |  |  | | |  | |  |  |
|  |  |  |  |  | |  | |  |  | | |  | |  |  |
| Age 45-64 (n=801) |  |  |  |  | |  | | Age 45-64 (n=431) |  | | |  | |  |  |
| Age |  | 1.043 (0.973 to 1.119) |  | 0.233 | |  | | Age | 1.048 (0.942 to 1.167) | | | 0.388 | |  |  |
| BMI |  | 1.078 (0.985 to 1.129) |  | 0.109 | |  | | BMI | 1.022 (0.924 to 1.113) | | | 0.648 | |  |  |
| Sex (Female) |  | 1.057 (0.711 to 3.292) |  | 0.109 | |  | | Sex (Female) | 0.422 (0.112 to 1.300) | | | 0.156 | |  |  |
| COPD |  | 2.283 (0.618 to 6.676) |  | 0.164 | |  | | HT | 2.644 (0.847 to 8.676) | | | 0.095 | |  |  |
| HT |  | 1.193 (0.513 to 2.614) |  | 0.669 | |  | | Severe DM | 2.367 (0.084 to 24.076) | | | 0.527 | |  |  |
| Severe DM |  | 1.895 (0.092 to 13.158) |  | 0.579 | |  | | CKD | 1.225 (0.048 to 12.315) | | | 0.878 | |  |  |
| CKD |  | 7.021 (1.364 to 27.959) |  | 0.009* | |  | | Not vaccinated | 2.842 (1.328 to 6.444) | | | 0.008* | |  |  |
| Solid tumor |  | 3.598 (0.500 to 15.753) |  | 0.129 | |  | |  |  | | |  | |  |  |
|  |  |  |  |  | |  | |  |  | | |  | |  |  |
| Age 65 or over (n=950) |  |  |  |  | |  | | Age 65 or over (n=631) |  | | |  | |  |  |
| Age |  | 0.997 (0.960 to 1.034) |  | 0.878 | |  | | Age | 0.916 (0.833 to 0.994) | | | 0.049* | |  |  |
| BMI |  | 1.087 (1.015 to 1.163) |  | 0.016* | |  | | BMI | 1.121 (0.979 to 1.274) | | | 0.087 | |  |  |
| Sex (Female) |  | 0.711 (0.399 to 1.267) |  | 0.246 | |  | | Sex (Female) | 1.072 (0.294 to 4.131) | | | 0.916 | |  |  |
| HT |  | 1.676 (0.943 to 2.978) |  | 0.077 | |  | | COPD | 2.179 (0.289 to 2.069) | | | 0.378 | |  |  |
| Severe DM |  | 0.167 (0.007 to 1.251) |  | 0.144 | |  | | HT | 3.324 (0.793 to 22.8166) | | | 0.140 | |  |  |
| CKD |  | 10.489 (2.186 to 48.692) |  | 0.002* | |  | | Severe DM | 1.070 (0.050 to 7.712) | | | 0.954 | |  |  |
| Solid tumor |  | 1.660 (0.206 to 7.509) |  | 0.566 | |  | | CKD | 2.727 (0.330 to 14.689) | | | 0.283 | |  |  |
|  |  |  |  |  | |  | | Solid tumor | 1.627 (0.083 to 10.141) | | | 0.661 | |  |  |
|  |  |  |  |  | |  | | Not vaccinated | 1.360 (0.565 to 2.961) | | | 0.456 | |  |  |
| *p<0.05 |  |  |  |  | |  |  |  |  | | |  |  | |  |

Abbreviations: OR, odds ratio; COPD, chronic obstructive pulmonary disease; BMI, body mass index; HT, hypertension; DM, diabetes mellitus; CKD, chronic kidney disease
