# Supplementary material for: Clinical trends among patients with asthma hospitalized for COVID-19 based on data from a nationwide database: an observational study
Source: BMC Pulm Med. 2024 Mar 2;24:105. doi: 10.1186/s12890-024-02917-x (PMC10909272; doi:10.1186/s12890-024-02917-x)
Supplement: Supplementary file 3 — Additional file 3: Table S3. Analysis of patients with asthma for occurrence of death. Abbreviations: COPD, chronic obstructive pulmonary disease; BMI, body mass index; HT, hypertension; DM, diabetes mellitus; CKD, chronic kidney disease. [file 12890_2024_2917_MOESM3_ESM.docx]

**TableS3: Asthma patients analysis (death)**

| Variable |  | Jan, 2020 – Jun, 2021 | | | Variable | | | | | Jul, 2021 – Dec, 2022 | | | |  |  |
| --- | --- | --- | --- | --- | --- | --- | --- | --- | --- | --- | --- | --- | --- | --- | --- |
|  |  | OR (95%CI) | P value | |  | | | | |  | OR (95%CI) | P value | |  |  |
| Age 18-44 (n=649) |  |  |  |  | |  | |  |  | | |  | |  |  |
| Age |  | 0.991 (0.944 to 1.042) |  | 0.729 | |  | |  |  | | |  | |  |  |
| BMI |  | 0.946 (0.865 to 1.019) |  | 0.184 | |  | |  |  | | |  | |  |  |
| Sex (Female) |  | 1.619 (0.753 to 3.605) |  | 0.2235 | |  | |  |  | | |  | |  |  |
| HT |  | 2.152 (0.986 to 4.645) |  | 0.050 | |  | |  |  | | |  | |  |  |
|  |  |  |  |  | |  | |  |  | | |  | |  |  |
| Age 45-64 (n=801) |  |  |  |  | |  | | Age 45-64 (n=431) |  | | |  | |  |  |
| Age |  | 1.153 (1.048 to 1.283) |  | 0.005* | |  | | Age | 1.094 (0.938 to 1.288) | | | 0.254 | |  |  |
| BMI |  | 1.150 (1.046 to 1.259) |  | 0.002* | |  | | BMI | 1.090 (0.965 to 1.212) | | | 0.127 | |  |  |
| Sex (Female) |  | 0.527 (0.170 to 1.472) |  | 0.237 | |  | | Sex (Female) | 0.166 (0.008 to 0.998) | | | 0.101 | |  |  |
| COPD |  | 4.939 (1.185 to 17.483) |  | 0.017* | |  | | HT | 0.545 (0.069 to 2.865) | | | 0.502 | |  |  |
| HT |  | 1.164 (0.366 to 3.095) |  | 0.851 | |  | | Severe DM | 3.946 (0.078 to 83.104) | | | 0.429 | |  |  |
| Severe DM |  | 2.911 (0.137 to 23.163) |  | 0.370 | |  | | CKD | 4.857 (0.111 to 110.516) | | | 0.345 | |  |  |
| CKD |  | 36.786 (5.785 to 213.974) |  | <0.001* | |  | | Solid tumor | 2.879 (0.121 to 28.232) | | | 0.412 | |  |  |
| Solid tumor |  | 4.159 (0.446 to 26.027) |  | 0.159 | |  | | Not vaccinated | 4.893 (1.590 to 19.063) | | | 0.010* | |  |  |
|  |  |  |  |  | |  | |  |  | | |  | |  |  |
| Age 65 or over (n=950) |  |  |  |  | |  | | Age 65 or over (n=631) |  | | |  | |  |  |
| Age |  | 0.994 (0.946 to 1.042) |  | 0.797 | |  | | Age | 1.010 (0.965 to 1.057) | | | 0.6657 | |  |  |
| BMI |  | 0.931 (0.834 to 1.031) |  | 0.186 | |  | | BMI | 0.955 (0.869 to 1.044) | | | 0.321 | |  |  |
| Sex (Female) |  | 1.058 (0.477 to 2.422) |  | 0.890 | |  | | Sex (Female) | 0.809 (0.367 to 1.797) | | | 0.598 | |  |  |
| COPD |  | 4.226 (1.052 to 13.201) |  | 0.022* | |  | | COPD | 1.538 (0.517 to 4.039) | | | 0.405 | |  |  |
| HT |  | 1.520 (0.684 to 3.381) |  | 0.299 | |  | | HT | 1.917 (0.861 to 4.599) | | | 0.124 | |  |  |
| Severe DM |  | 1.450 (0.150 to 8.066) |  | 0.709 | |  | | CKD | 0.580 (0.031 to 3.083) | | | 0.608 | |  |  |
| CKD |  | 7.949 (1.179 to 43.820) |  | 0.022* | |  | | Solid tumor | 1.450 (0.321 to 4.642) | | | 0.574 | |  |  |
| Solid tumor |  | 2.557 (0.292 to 12.134) |  | 0.305 | |  | | Not vaccinated | 1.132 (0.679 to 1.794) | | | 0.613 | |  |  |
| *p<0.05 |  |  |  |  | |  |  |  |  | | |  |  | |  |

Abbreviations: OR, odds ratio; COPD, chronic obstructive pulmonary disease; BMI, body mass index; HT, hypertension; DM, diabetes mellitus; CKD, chronic kidney disease
